# A novel dedicated transparent hood facilitating cholangioscopy-guided lithotripsy in a patient with choledocholithiasis

**DOI:** 10.1055/a-2987-8996

**Published:** 2026-11-05

**Authors:** Ryuichi Watanabe, Yuki Tanisaka, Shomei Ryozawa, Masafumi Mizuide, Akashi Fujita, Suguru Ito, Ryosuke Hamamura

**Affiliations:** 1 Gastroenterology 183786 Saitama Medical University International Medical CenterHidakaJapan; 2 GastroenterologySaitama Medical University International Medical CenterHidakaSaitamaJapan; 3 Department of Gastroenterology 183786 Saitama Medical University International Medical CenterHidakaJapan

## A Case Description



**Video 1** Successful cholangioscopy-guided lithotripsy using a novel dedicated transparent hood for cholangioscopy.


Endoscopic stone extraction is the first-line treatment for choledocholithiasis, and complete stone clearance can be achieved in most cases. 1​ However, stone extraction may be challenging in patients with large or multiple stones, particularly when stones are located proximal to a biliary stricture. Cholangioscopy-guided lithotripsy is effective for the treatment of difficult bile duct stones. 2​ 3​ ​4​ Nevertheless, depending on the location and orientation of the stone, achieving adequate contact between the lithotripsy probe and the stone may be technically difficult. We report a case in which a novel dedicated transparent hood for cholangioscopy facilitated cholangioscopy-guided lithotripsy for difficult choledocholithiasis.

A 70-year-old man was referred to our institution for treatment of choledocholithiasis. Computed tomography revealed a common bile duct stone located proximal to a biliary stricture ( Fig. 1 ). Endoscopic retrograde cholangiopancreatography was performed for stone extraction ( Video 1 ). Cholangiography revealed the presence of the stone proximal to the biliary stricture ( Fig. 2 ). Because fluoroscopy-guided stone extraction was considered technically difficult, cholangioscopy-guided lithotripsy was attempted using a 9-Fr cholangioscope (eyeMAX; Micro-Tech, China). 5​ Cholangioscopy revealed the stone immediately proximal to the stricture. However, because the stone was visualized tangentially, achieving stable probe-to-stone contact for lithotripsy was considered technically difficult ( Fig. 3 ). To facilitate cholangioscopy-guided lithotripsy, a dedicated transparent hood (eyeMAX Disposable Distal Cap; Micro-Tech, China) was attached to the tip of the cholangioscope ( Fig. 4a, b ). The hood enabled en face visualization of the stone immediately proximal to the stricture and facilitated stable probe-to-stone contact, thereby allowing efficient stone fragmentation ( Fig. 4c, d ). Complete stone extraction was subsequently achieved ( Fig. 5 ).

**Fig. 1:**
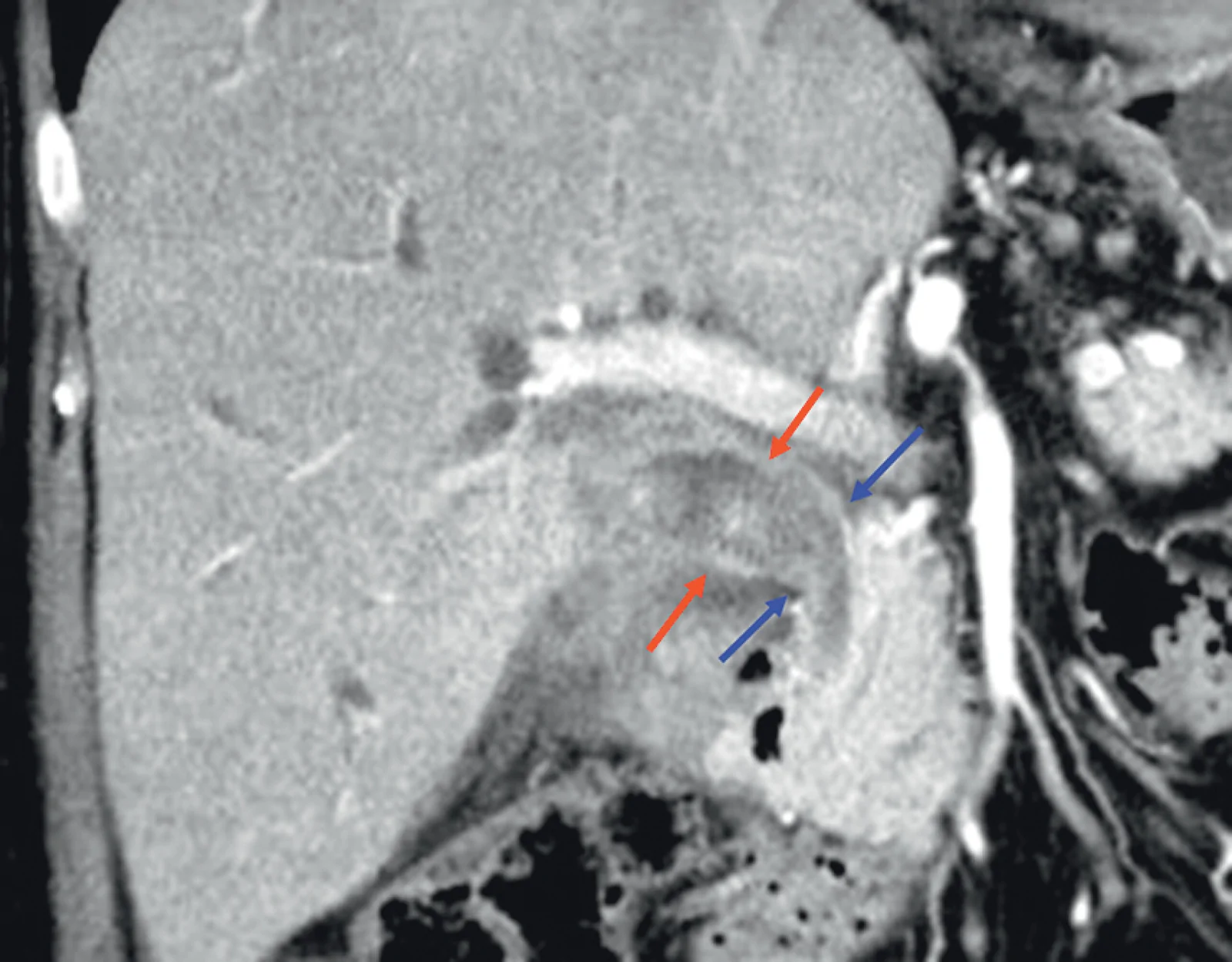
Computed tomography revealing a common bile duct stone (red arrow) located proximal to a biliary stricture (blue arrow).

**Fig. 2:**
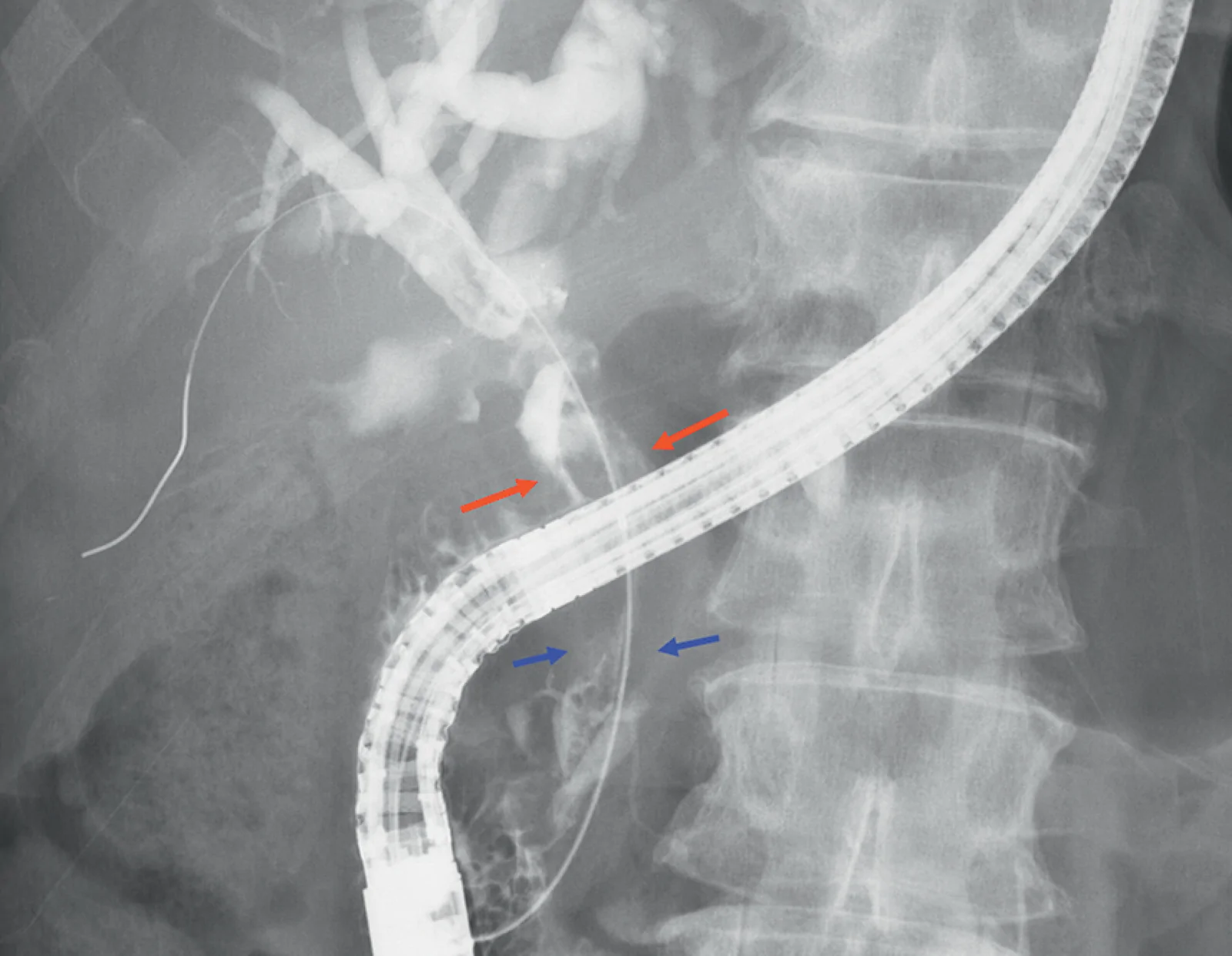
Cholangiography revealing the presence of the stone (red arrow) proximal to the biliary stricture (blue arrow).

**Fig. 3:**
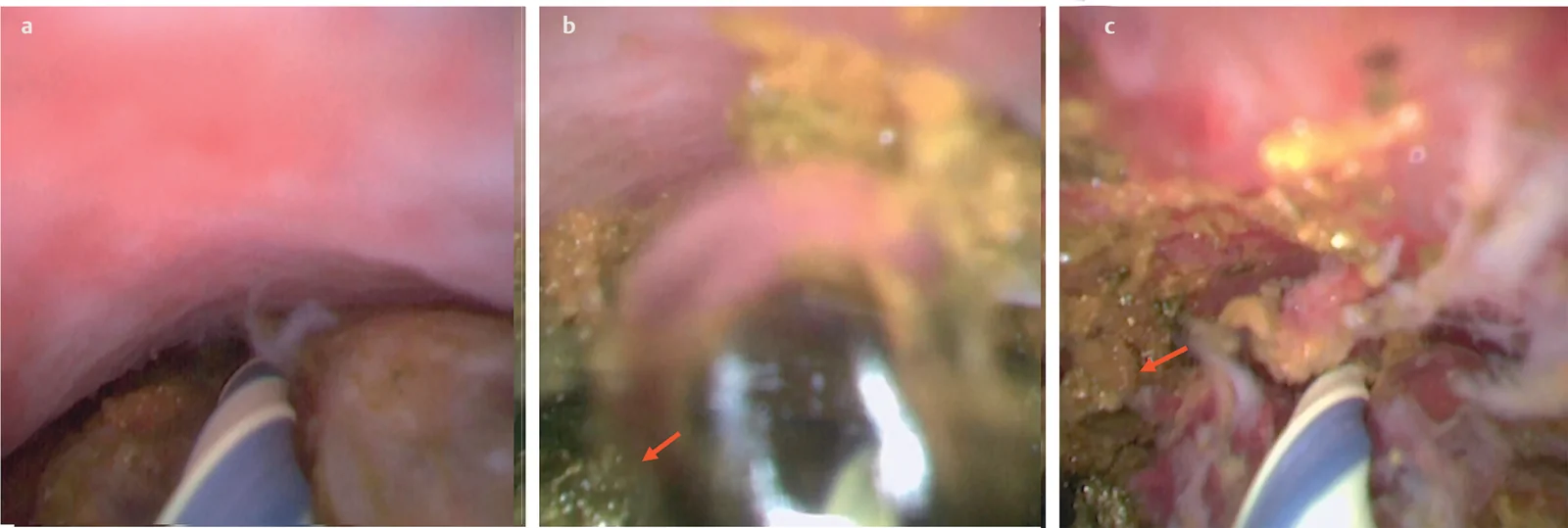
Cholangioscopic findings. ( **a** ) Cholangioscopy revealing a biliary stricture in the common bile duct. ( **b** and **c** ) Cholangioscopy revealing the stone immediately proximal to the stricture, visualized tangentially (red arrow).

**Fig. 4:**
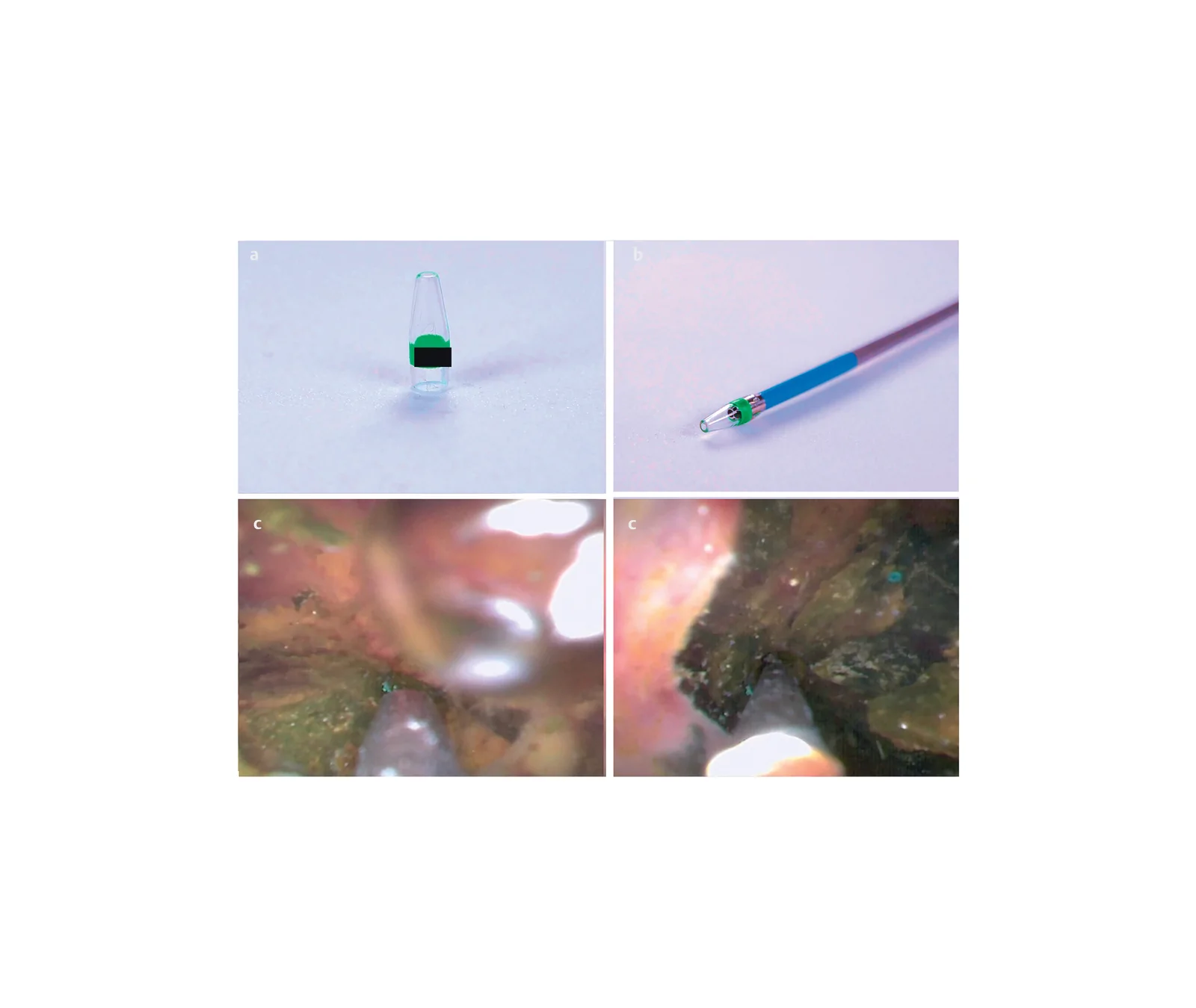
Cholangioscopic findings using a dedicated transparent hood. ( **a** and **b** ) A dedicated hood (eyeMAX Disposable Distal Cap; Micro-Tech, China) attached to the tip of the cholangioscope. ( **c** and **d** ) The hood enabled en face visualization of the stone immediately proximal to the stricture and facilitated stable probe-to-stone contact, thereby allowing efficient stone fragmentation.

**Fig. 5:**
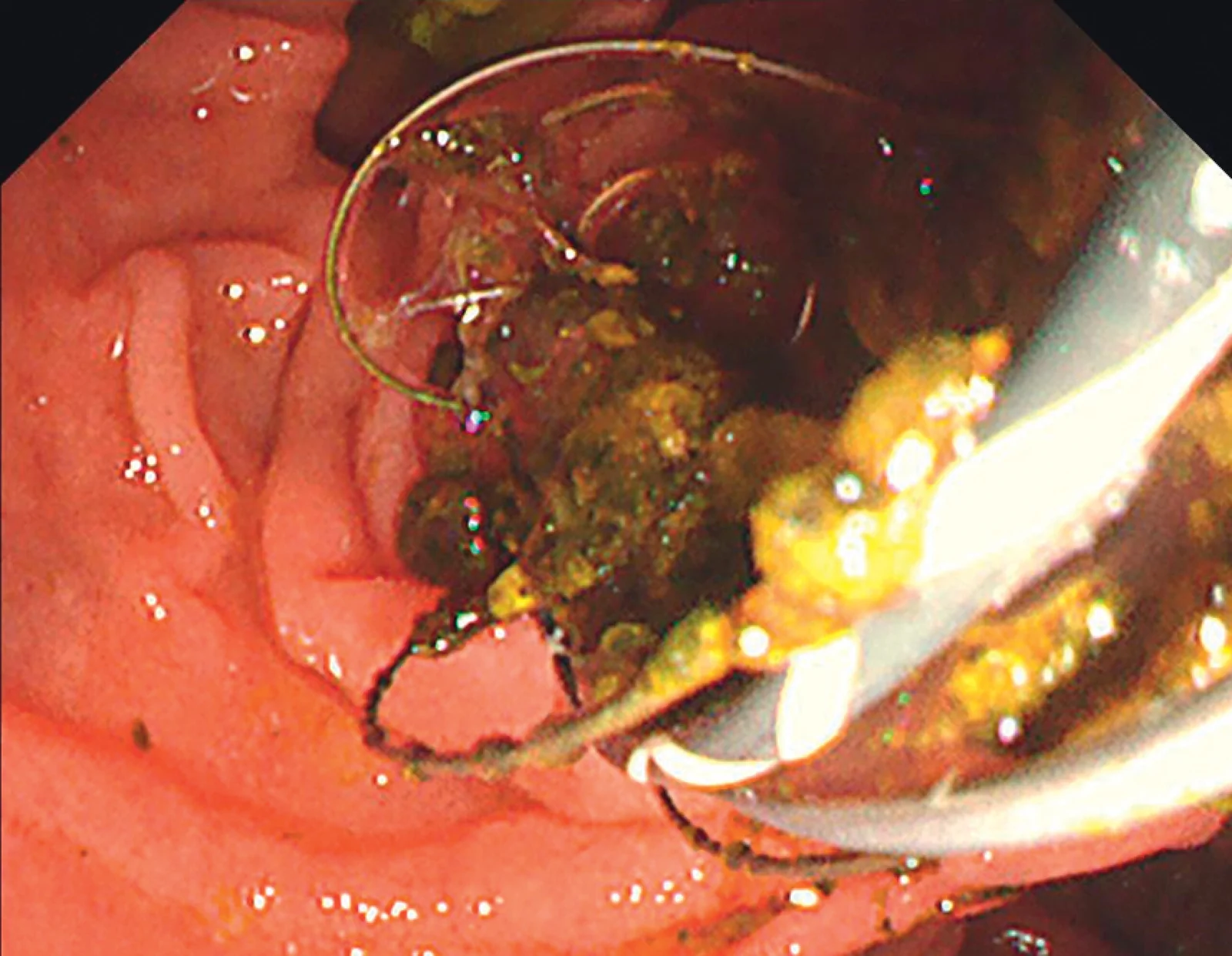
An endoscopic image showing successful complete stone extraction.

This novel accessory may improve visualization and probe-to-stone contact during cholangioscopy-guided lithotripsy, facilitating the treatment of difficult bile duct stones when tangential visualization hinders effective lithotripsy.

Endoscopy_UCTN_Code_TTT_1AR_2AH

## Declaration of GenAI Use

ChatGPT (OpenAI) was used solely to improve the English language and grammar of the manuscript. It was not used to generate scientific content, analyze data, or draw conclusions. The authors reviewed and approved the final manuscript and take full responsibility for its content.

## References

[1] Manes G Paspatis G Aabakken L Endoscopic management of common bile duct stones: European Society of Gastrointestinal Endoscopy (ESGE) guideline Endoscopy 2019 51 05 472 491 10.1055/a-0862-0346 30943551

[2] Veld J V van Huijgevoort NC M Boermeester M A A systematic review of advanced endoscopy-assisted lithotripsy for retained biliary tract stones: laser, electrohydraulic or extracorporeal shock wave Endoscopy 2018 50 09 896 909 10.1055/a-0637-8806 29991072

[3] Maydeo A P Rerknimitr R Lau J Y Cholangioscopy-guided lithotripsy for difficult bile duct stone clearance in a single session of ERCP: results from a large multinational registry demonstrate high success rates Endoscopy 2019 51 10 922 929 10.1055/a-0942-9336 31250408

[4] McCarty T R Gulati R Rustagi T Efficacy and safety of peroral cholangioscopy with intraductal lithotripsy for difficult biliary stones: a systematic review and meta-analysis Endoscopy 2021 53 02 110 122 10.1055/a-1200-8064 32544959

[5] Tanisaka Y Ryozawa S Mizuide M Peroral cholangioscopy-guided laser lithotripsy using a thin cholangioscope under balloon enteroscopy for Roux-en-Y anastomosis Endoscopy 2025 57S 01E1315 E1317 10.1055/a-2738-7298 PMC12629838 41260579

